# Environmental Electronic Vape Exposure from Four Different Generations of Electronic Cigarettes: Airborne Particulate Matter Levels

**DOI:** 10.3390/ijerph15102172

**Published:** 2018-10-03

**Authors:** Carmela Protano, Pasquale Avino, Maurizio Manigrasso, Valerio Vivaldi, Franco Perna, Federica Valeriani, Matteo Vitali

**Affiliations:** 1 Department of Public Health and Infectious Diseases, Sapienza University of Rome, P.le Aldo Moro, 5, 00185 Rome, Italy; carmela.protano@uniroma1.it (C.P.); valerio-vivaldi@hotmail.it (V.V.); francopianoman@hotmail.it (F.P.); matteo.vitali@uniroma1.it (M.V.); 2 Department of Agricultural, Environmental and Food Sciences (DiAAA), University of Molise, via De Sanctis, I-86100 Campobasso, Italy; 3 Department of Technological Innovations, INAIL, Via IV Novembre 144, 00187 Rome, Italy; m.manigrasso@inail.it; 4 Department of Movement, Human and Health Sciences, University of Rome “ForoItalico”, Piazza Lauro De Bosis 6, 00135 Rome, Italy; federica.valeriani@uniroma4.it

**Keywords:** electronic cigarettes, environmental electronic vape exposure, particulate matter

## Abstract

Electronic cigarettes (e-cigs) were introduced into the market in 2006 and their technological features have evolved substantially over time. Currently, there are four different generations of e-cigs that are broadly considered less harmful than the use of combusted tobacco products although passive exposure to aerosols often occurs in public spaces and indoor environments. The study aim was to evaluate the levels of airborne particulate matter (PM) emitted during the use of all the four generations of e-cigs, testing different use modalities. PM_10_, PM_4_, PM_2.5_ and PM_1_ were measured through a Dusttrak ™ II Aerosol Monitor, for a total of 20 independent experiments. All tested e-cigs devices produced PM during their use, and PM_10_ was almost made of PM_1_ size fraction. In addition, we observed a progressive increase in PM emission from the first to the fourth generation, and an upward trend of PM_1_ emitted by the fourth generation e-cig with an increase in the operating power. The results showed that, whatever the model adopted, passive vaping does occur. This finding supports the need for legislative interventions to regulate the e-cigs use in public places and other enclosed environments, in order to protect the health of any subject who is potentially exposed.

## 1. Introduction

Electronic cigarettes (e-cigs) are electric devices which have become very popular worldwide in the last decade [1,2]. Their popularity among the smokers’ community has grown exponentially, so that e-cigs have become a leading product among the various alternatives to traditional cigarettes. Smokers use e-cigs to quit, but also to experience new ways of smoking or to avoid smoking bans. During the last decade, the use of e-cigs also has spread among non-smokers [3,4,5]. E-cig design has evolved from the earliest generation of “cig-a-like”, to the recently commercialized “fourth” generation. The latest models can be vaped with sub-ohm resistance at high wattage and, consequently, can release larger amounts of aerosols than the older devices [6,7]. Therefore, a main concern is related to the wide diffusion of e-cig smokers and the potential impact on passively exposed subjects.

The evaluation of health risks associated to e-cig smoking depends firstly on the safety of the liquid formulation mix and of its single chemical components [8,9] and several types of accidents can happen as well, including those due to explosion of the e-cigarette battery. The basic components of e-liquids are propylene glycol and vegetable glycerin, to which nicotine and flavorings are added in different concentrations. Regardless of the already studied chemical risks, the safety of these substances when vaporized is still under evaluation and an accurate risk assessment is strongly limited by the lack of epidemiological data and by the heterogeneous set of flavoring substances widely available on the market [10]. The possible toxicity of the different products emitted during vaping, draws the attention to Environmental Electronic Vape (EEV) exposure. In our previous studies [11,12,13], we already demonstrated that passive exposure to PM occurs when using second generation e-cigs. This finding is of particular concern for public health because PM is a known risk factor for different diseases including pulmonary [14], cardiovascular [15], neurodegenerative disorders [16] or cancers [17], independently from its composition. Adverse health effects have been mainly linked to the PM_10_ size fraction, and to fractions with lower aerodynamic diameters. In this study, we considered all the four generations of e-cigs currently available and evaluated the levels of different sizes of PM (sizes ≤10 or PM_10_, ≤4 or PM_4_, ≤2.5 or PM_2.5_ and ≤1 μm or PM_1_), testing different e-liquids and different “mods” of use.

## 2. Background

Currently, four different generations of e-cigs are available on the market [18]. The first generation is about the same size but heavier than a conventional cigarette; it is often called “cig-a-like”. At the end of the device, a LED light that lights up upon inhalation is present, allowing one to monitor its use.

The second generation, or “personal vaporizer”, usually has the appearance of a pen or a laser pointer, and is considerably larger than a cig-a-like. When using a second-generation e-cig, the “fire” button must be manually pressed during inhalation. Besides, it also has much larger capacity battery (mostly three to seven times larger, from 450 to 1100 mAh) than the cig-a-like one, which means that, in most cases, it can provide vaping power for longer periods, about 1–2days. Some medium-sized e-cigs can also allow the user to adjust the voltage with the help of two small buttons or by rotating the base of the battery, providing an additional control for the user.

The third generation is available in several different sizes and shapes. In most cases, however, it is considerably larger than the second generation or cig-a-like ones. Almost all the third generation e-cigs are equipped with a manual “fire” button. The main element is a “mod”, the source of energy. There are two types of mod: (1) mechanical mod, a very simple device without electronic circuits that has only a fire button, a battery compartment and a connector; (2) regulated mod, that presents hardware that allows the vaper to change the voltage and/or the output in watts.

The fourth generation is the most recent developed, powerful, advanced and innovative device on the market. Together with the hardware for changing the voltage and/or the output in watts, it presents also mods with automatic temperature control and the ability to manage very low resistances (sub-ohm).

## 3. Materials and Methods

### 3.1. Experimental Tests

PM_10_, PM_4_, PM_2.5_ and PM_1_ emitted by the four main generations of e-cigs were measured. The devices and e-liquids used, together with the description of the 20 tested operative conditions and the relative codes, are reported in Table 1.

We considered a nicotine concentration range including those typically used by vapers. The electrical parameters adopted were within the settings allowed by the e-cigs models studied and commonly adopted by different vapers.

The experiments and PM measurements were performed in a room of 52.7 m^3^, in which the window and the door were closed; during the measurements, the temperature and the relative humidity were between 24 and 26 °C and 25 and 32%. Voluntary smokers (already formerly smokers) were employed at University of Rome La Sapienza. The study was not sponsored and was approved by the Local Ethics Committee (Policlinico Umberto I/University of Rome La Sapienza, protocol code n. 3520). The air exchange rate (λ) was calculated using the tracer gas technique, as previously reported [11].

The aerosol concentrations for each size fraction (PM_10_, PM_4_, PM_2.5_, PM_1_), expressed in μg m^−3^, were measured in “cumulative” mode, that is including the mass of all particles that are smaller than or equal to the defined size. Measurements were performed by means of a portable, laser-operated aerosol mass analyzer (Dusttrak ™ II Aerosol Monitor, model 8530, TSI, 0.1–10 µm particle size range) [19]. The aerosol was sampled directly through the entry of the instrument without using any tube. The instrument was placed approximately 1.5 m above the floor level and approximately 1.5 m from the e-cig vaper, thus simulating the breathing zone of a passive exposed subject. For each experiment, the measurement was performed from five minutes before until one hour after the end of the vaping session. 12 puffs were made for each session lasting about 5.5 min (1 puff about each thirty seconds), since the common way of smoking typically consists of 10–12 puffs of a cigarette, for a period of about 5–6 min [12]. All the tests were repeated in triplicate. After each experiment, the door and window were opened to rebalance the room atmosphere. Since the rebalance is related to several variable factors (temperature difference, ventilation, outdoor wind speed, indoor humidity, etc.), doors and windows were opened overnight.

### 3.2. Statistical Elaboration

**Figure 1:**
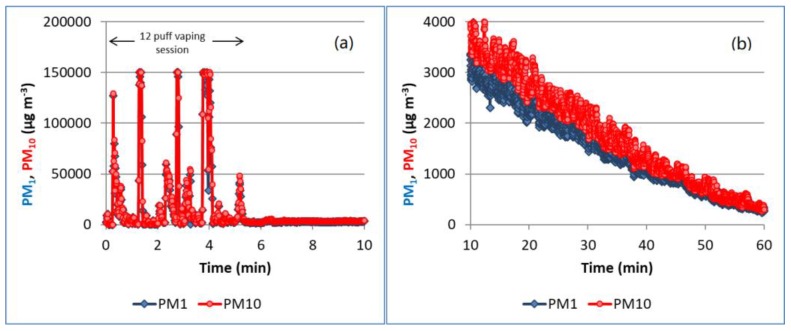
Comparison between the levels of PM_1_ and PM_10_ released during the vaping session of 4e-cig_0.15Ω-150W_N (**a**) and the following decay phase (**b**).

Statistical analyses were carried out using IBM-SPSS Statistics for Windows, Version 25.0 (2017, IBM Corp.: Armonk, NY, USA). Statistical elaborations were performed on PM_1_ concentrations, because PM_10_ was almost made of PM_1_ size fraction in all the experiments performed. As a demonstration, Figure 1 shows the PM_1_ and PM_10_ concentrations temporary trends during and after the puffing sessions for the 4e-cig_0.15Ω-150W_N test.

Mann-Whitney U test was used for assessing differences in median PM_1_ levels measured respectively before and during the vaping session for each test. Kruskal-Wallis test was used with pairwise post-hoc tests in order to evaluate differences in median PM_1_ levels measured during each vaping session of all experiments.

## 4. Results

**Figure 2:**
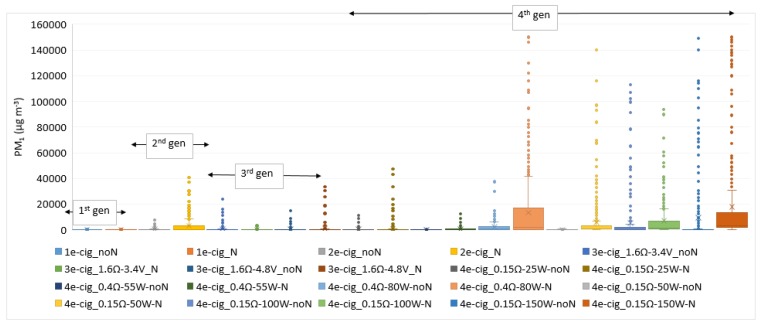
PM_1_ concentrations (μg m^−3^) during all monitored vaping sessions.

Table 2 shows summary statistics of PM_1_ levels before and during the vaping session for each test. The results reported in Table 2 evidence, in all cases, a significant increase in PM_1_ concentration during the vaping session compared to the levels measured before starting vaping (*p*-value always <0.001). PM_1_ levels during the vaping sessions widely varied according to the device tested, ranging from a median value of 17.00 μgm^−3^ during the use of 3e-cig_1.6Ω-3.4V_N to 2895.00 μg m^−3^ during the use of 4e-cig_0.15Ω-150W_N. Figure 2 shows the boxplots of PM_1_ levels during all the vaping sessions that were considered. Table 3 shows the pairwise post-hoc tests performed together with the Kruskal-Wallis test.

**Figure 3:**
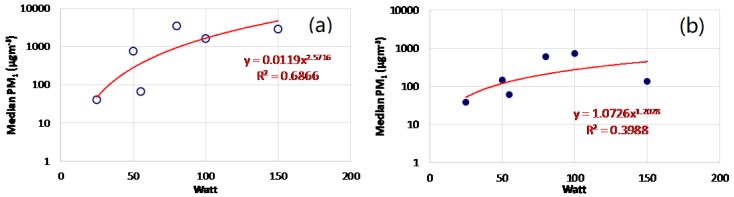
PM_1_ concentrations (μg m^−3^) emitted by the fourth generation e-cig as a function of operating power (Watt), both for e-liquid with nicotine (**a**) and e-liquid nicotine-free (**b**).

Figure 2 shows an increase in the PM_1_ levels from the first to the fourth generation of tested e-cigs; in addition, the mod setting is an influencing parameter. Indeed, considering the third and the fourth generations of e-cigs, we found a great variability of PM_1_ concentrations among the different experiments, as evidenced in Table 2 too. Pairwise post-hoc tests (Table 3) demonstrated that the great part (tests from 12 to 20) of the operating modes considered for the fourth generation e-cig, which was determines an emission of PM_1_ levels significantly higher than those of other generations. Moreover, PM_1_ concentrations emitted by 1e-cig_N and 2e-cig_N differ significantly from all the other tests. Figure 3 shows the upward trend of PM_1_ emitted by the fourth generation e-cig with the increase in the operating power (Watt) (R^2^ equal to 0.69 and 0.40 for test performed with nicotine and nicotine-free e-liquids, respectively).

## 5. Discussion

To date, the use of e-cigs has significantly increased compared to 2006, which was the year of their entry on the market. However, the safety of e-cigs is still under evaluation for both vapers and subjects exposed to EEV. We evidenced in a previous study [13] that EEV exposure does occur when a second generation e-cig is smoked in an indoor environment.

In the light of the different generations of e-cigs released in commerce, the results of the present study add some relevant new insights on the issue of EEV exposure. Firstly, all generations of e-cigs produce PM during their use; thus, whatever the model adopted, passive vaping does occur. This finding agrees with previous studies on passive vaping and indoor PM concentrations [20,21]. In particular, Czogala et al. [20] monitored different vaping conditions (both with smoking machine and volunteers ad libitum vaping) and reported a mean PM_2.5_ concentration equal to 33.1 µg m^−3^ for smoking machine and 151.7 µg m^−3^ for volunteer vapers, respectively. Besides, it is important to note that all the e-cigs tested in this study at the various setting modes emitted a median PM_1_ concentration higher than the limits recommended by the World Health Organization in terms of 24-h mean concentrations for greater PM size fractions (25 µgm^−3^ for PM_2.5_ and 50 µg m^−3^ for PM_10_) [22]. This is of particular concern for infants and children more than for adults, because they receive the highest aerosol doses per kg bw [12]. Furthermore, it is important to note that the new generation of e-cigs can be vaped at high wattage and, consequently, release greater amounts of aerosol than the older devices did.

The data evidence both a nicotine effect and a power effect on the amount of aerosol released. The liquids with nicotine produced statistically significant PM levels higher than the nicotine free liquids (keeping constant the electrical parameters). There were only two exceptions: the first one represented by 3e-cig_1.6Ω-4.8V (higher PM concentration for nicotine liquid, but not statistically significant), the second one represented by 3e-cig_1.6Ω-3.4V (statistically significant higher PM concentrations for nicotine free liquids). Such exceptions may be explained by the possible different vaping topography (puff duration, breath-hold, exhale time) adopted by the volunteers. The use of a smoking machine would have overridden this drawback but would have produced an exposure pattern different from those currently encountered in real indoor settings.

As to the power effect, we observed a general increment of PM emission from the first to the fourth generation (Figure 2) with increasing the operating wattage. It is important to underline that, in several tests, the levels of PM_1_ released by e-cigs reached median concentrations exceeding those generated by traditional cigarettes (mean value of PM_1_ equal to 1544.0 µg m^-3^ during smoking a traditional cigarette) [23].

For a comprehensive health evaluation not only PM levels are relevant, but also the chemical composition of the released aerosol. A recent systematic review [24] highlighted that the composition of the aerosol emitted during the use of e-cigs is distinctly different from that emitted by traditional cigarettes. It indicates that the e-cigs emissions contain, in addition to PM, toxic compounds such as carbonyls, metals and volatile organic compounds, let alone nicotine. These compounds are generally at lower concentrations than those found in passive traditional cigarette smoke. Williams et al. [25,26] analyzed e-cigs aerosol, detecting inorganic and metal compounds, including toxic metals such as nickel, zinc and silver.

Besides, more recent studies addressed the vapor phase emissions of sub-ohm operated fourth generations e-cigs [7,27,28]. Most remarkably, Talih et al. [7] found that volatile aldehyde emissions increase in a correlated way as a function of the power per coil surface area.

In addition to the presence of toxicologically relevant components in e-cigs liquids and aerosol, the results discussed here should be regarded also in the context of the scientific evidences and biological plausibility for a possible impact on human health. In particular, negative outcomes due to e-cigs liquids have been reported in terms of alveolar macrophage dysfunction, expression of phagocytic recognition receptors and cytokine secretion pathways [29]. Coherently, an increased susceptibility to pneumococcal [30] and to viral [31] infections has also been evidenced by in vitro studies. Moreover, e-liquids were found to be cytotoxic and, in some cases, caused a reduction in cell viability and DNA damage. Therefore, their mutagenicity for the mucosal tissue of the upper aerodigestive tract and their effects on head and neck cancer cannot be excluded [32]. It is worth observing that all these findings have been demonstrated both for nicotine and nicotine-free e-cig liquids. In particular, some specific components, namely glycerin and propylene glycol, alter the mediator activities involved in the inflammation and several flavors were shown to induce cytotoxic effects [33].

The main limitations in the present study include, first of all, the presence of confounders. The great number of variables makes difficult to fully standardize and monitor the exposure conditions, due to the different e-cigs models, available e-liquids or vaping conditions used by different subjects. We did not apply a smoking machine for the experiments, but considered several vaping sessions performed by voluntary vapers, simulating real scenarios. Therefore, we took into account that a portion of the aerosol released by the e-cigs was deposited in the respiratory system and not dispersed in the room. On the other hand, this implies that the aerosol emissions may to some extent vary from individual to individual due to the different vaping patterns in different subjects. Another limitation is represented by the limited number of the tested devices and experiments, which were performed in the present pilot study. Even if our study can mimic exposure conditions in real life, and even if provided significant reproducible data, further investigations are required to deepen the knowledge on these topics and provide a larger pattern of exposure conditions. Additional information is needed to unravel the possible health impact of different e-cigs and liquids under different exposure conditions. Moreover, new tests to measure size number distributions of the aerosol released by the same devices are to be performed and, based on them, a dosimetry evaluation of passively exposed subjects should be carried out.

## 6. Conclusions

The present study adds new data demonstrating that EEV exposure to PM does occur during the use of all available e-cigs models up to the most recent fourth generation devices. This result evidences the need for new investigations to evaluate the size distributions of the aerosol emitted, with particular emphasis on nanoparticles and on the relevant doses potentially deposited into the respiratory system of passively exposed individuals.

The findings are relevant from a public health point of view, supporting with experimental data the need for specific legislative interventions to regulate the use of these devices in public places and other indoor environments, in order to protect the health of subject exposed to EEV. Particular attention should be dedicated to the public environments in which more susceptible or fragile individuals can be present, such as schools and hospitals. Our data can support educational interventions aimed to increase the awareness on the threats due to the use of e-cigs. It is important to discourage the use of e-cigs also in private environments, suggesting healthy behaviors and preventing exposure on population of different age and health conditions. The actions of law already adopted for preventing environmental tobacco smoke exposure should be considered and extended to EEV exposure.

## Figures and Tables

**Table 1:** E-cigs, e-liquids, operative conditions and relative codes of the experiments.

E-Cig Generation	E-Cig Types	Liquid Characteristics	Resistance (Ω), Voltage (V), Wattage (W) Conditions	E-Cig Code
First	Young Category^®^, an e-cig with disposable filter, integrated atomizer and cotton already soaked in liquid	Liquidwithout nicotine	*	1e-cig_noN
		Liquidwith a nicotine at 24 mg mL^−1^	*	1e-cig_N
Second	Smooke^®^, an e-cig consisting of a high capacity rechargeable battery, with a separate tank	Flavourart Dark Vapure Heaven Juice^®^ without nicotine	*	2e-cig_noN
		Flavourart Dark Vapure Heaven Juice^®^ with nicotine at 18 mg mL^−1^	*	2e-cig_N
Third	JustFog Q16 Kit^®^, consisting of a JustFog J-Easy 9^®^ battery and a JustFog Q 16^®^ atomizer (1.6 Ohm). The battery has a capacity of 900 mAh and allows the regulation of the voltage, from 3.4 V to 4.8 V	Dea Velvet^®^ without nicotine	1.6Ω, 3.4V	3e-cig_1.6Ω-3.4V_noN
			1.6Ω, 4.8V	3e-cig_1.6Ω-4.8V_noN
		DeaFlavour Lady In Black^®^ with nicotine at 9 mg mL^−1^	1.6Ω, 3.4V	3e-cig_1.6Ω-3.4V_N
			1.6Ω, 4.8V	3e-cig_1.6Ω-4.8V_N
Fourth	G 150 Smok Kit^®^ equipped with V8 Baby-Q2 Smok atomizer^®^ (0.15 and 0.4 Ohm). The battery capacity was equal to 4200 mAh, the wattage variable from 1 to 150 W, and the resistance between 0.1 and 3 Ohms	Pacha Mama—Mango Pitaya Ananas^®^ without nicotine	0.15Ω, 25W	4e-cig_0.15Ω-25W_noN
			0.4Ω, 55W	4e-cig_0.4Ω-55W_noN
			0.4Ω, 80W	4e-cig_0.4Ω-80W_noN
			0.15Ω, 50W	4e-cig_0.15Ω-50W_noN
			0.15Ω, 100W	4e-cig_0.15Ω-100W_noN
			0.15Ω, 150W	4e-cig_0.15Ω-150W_noN
		SmookeKannel^®^ with nicotine at 9 mg mL^−1^	0.15Ω, 25W	4e-cig_0.15Ω-25W_N
			0.4Ω, 55W	4e-cig_0.4Ω-55W_N
			0.4Ω, 80W	4e-cig_0.4Ω-80W_N
			0.15Ω, 50W	4e-cig_0.15Ω-50W_N
			0.15Ω, 100W	4e-cig_0.15Ω-100W_N
			0.15Ω, 150W	4e-cig_0.15Ω-150W_N

* Resistance (Ω), voltage (V), wattage (W) conditions are not settable by the user and not declared by the producers.

**Table 2:** Summary statistics of PM_1_ concentrations (μg m^−3^) before and during the vaping session for each test.

E-cig Code	Before Vaping Session		During Vaping Session		*p*-Value
	AM [SD]	Median [IQR]	AM [SD]	Median [IQR]	
**1e-cig_noN**	41.27 [19.09]	38.00 [9.00]	79.69 [80.13]	64.00 [12.00]	<0.001
**1e-cig_N**	43.86 [18.75]	36.00 [8.00]	105.52 [117.10]	89.00 [10.00]	<0.001
**2e-cig_noN**	21.34 [7.67]	19.00 [4.00]	534.00 [1266.88]	34.50 [309.00]	<0.001
**2e-cig_N**	18.33 [6.74]	18.00 [2.00]	3428.85 [5857.54]	648.00 [4256.00]	<0.001
**3e-cig_1.6Ω-3.4V_noN**	21.56 [6.31]	21.00 [7.00]	789.48 [2300.46]	36.00 [192.00]	<0.001
**3e-cig_1.6Ω-3.4V_N**	26.22 [6.58]	25.00 [3.00]	54.39 [179.23]	17.00 [22.00]	<0.001
**3e-cig_1.6Ω-4.8V_noN**	21.45 [6.75]	19.00 [7.00]	522.29 [1729.70]	43.00 [99.00]	<0.001
**3e-cig_1.6Ω-4.8V_N**	26.22 [13.58]	25.00 [11.00]	1005.81 [4405.06]	22.00 [297.00]	<0.001
**4e-cig_0.15Ω-25W_noN**	20.96 [2.74]	20.00 [1.00]	384.53 [1327.67]	39.00 [99.00]	<0.001
**4e-cig_0.15Ω-25W_N**	35.44 [6.32]	34.00 [2.00]	963.24 [4605.46]	41.00 [96.00]	<0.001
**4e-cig_0.4Ω-55W_noN**	31.67 [8.79]	28.00 [5.00]	74.50 [40.70]	61.00 [29.00]	<0.001
**4e-cig_0.4Ω-55W_N**	43.87 [6.23]	43.00 [4.00]	472.93 [1181.44]	66.00 [322.00]	<0.001
**4e-cig_0.4Ω-80W_noN**	35.44 [6.32]	34.00 [2.00]	2238.34 [3931.00]	603.00 [2414.00]	<0.001
**4e-cig_0.4Ω-80W_N**	41.66 [7.36]	39.00 [6.00]	14,887.00 [25,725.24]	3475.00 [19,658.00]	<0.001
**4e-cig_0.15Ω-50W_noN**	41.27 [19.09]	38.00 [9.00]	177.69 [80.61]	144.00 [109.00]	<0.001
**4e-cig_0.15Ω-50W_N**	43.55 [7.725]	41.00 [7.00]	5949.16 [15,452.17]	766.00 [2483.00]	<0.001
**4e-cig_0.15Ω-100W_noN**	39.28 [17.21]	34.00 [7.00]	5637.34 [19,136.38]	732.00 [1769.00]	<0.001
**4e-cig_0.15Ω-100W_N**	43.55 [7.73]	41.00 [7.00]	2572.72 [4301.85]	1610.00 [1292.00]	<0.001
**4e-cig_0.15Ω-150W_noN**	41.27 [19.09]	38.00 [9.00]	12,925.34 [31,590.92]	136.00 [4619.00]	<0.001
**4e-cig_0.15Ω-150W_N**	44.67 [8.59]	40.00 [5.00]	14,640.47 [32,776.91]	2895.00 [4373.00]	<0.001

**Table 3:** Pairwise post-hoc tests carried out considering each monitored setting mod.

	E-Cig Code	2	3	4	5	6	7	8	9	10	11	12	13	14	15	16	17	18	19	20
1	1e-cig_noN	*	ns	*	ns	*	ns	ns	ns	*	*	*	*	*	*	*	*	*	*	*
2	1e-cig_N		*	*	*	*	*	*	*	*	*	*	*	*	*	*	*	*	*	*
3	2e-cig_noN			*	ns	*	ns	ns	ns	ns	ns	*	*	*	*	*	*	*	*	*
4	2e-cig_N				*	*	*	*	*	*	*	*	*	*	*	*	*	*	*	*
5	3e-cig_1.6Ω-3.4V_N					ns	ns	ns	ns	*	ns	*	*	*	*	*	*	*	*	*
6	3e-cig_1.6Ω-3.4V_noN						ns	*	*	ns	ns	*	*	*	*	*	*	*	*	*
7	3e-cig_1.6Ω-4.8V_noN							ns	ns	ns	ns	*	*	*	*	*	*	*	*	*
8	3e-cig_1.6Ω-4.8V_N								ns	ns	ns	*	*	*	*	*	*	*	*	*
9	4e-cig_0.15Ω-25W_noN									*	*	*	*	*	*	*	*	*	*	*
10	4e-cig_0.15Ω-25W_N										*	*	*	*	*	*	*	*	*	*
11	4e-cig_0.4Ω-55W_noN											*	*	*	*	*	*	*	*	*
12	4e-cig_0.4Ω-55W_N												*	*	*	*	*	*	*	*
13	4e-cig_0.4Ω-80W_noN													*	*	*	*	*	*	*
14	4e-cig_0.4Ω-80W_N														*	*	*	*	*	*
15	4e-cig_0.15Ω-50W_noN															*	*	*	*	*
16	4e-cig_0.15Ω-50W_N																*	*	*	*
17	4e-cig_0.15Ω-100W_noN																	*	*	*
18	4e-cig_0.15Ω-100W_N																		*	*
19	4e-cig_0.15Ω-150W_noN																			*
20	4e-cig_0.15Ω-150W_N																			

Notes: ns = not significant; * *p*-value < 0.05.
